# Prognostic factors for inpatient functional recovery following total hip and knee arthroplasty: a systematic review

**DOI:** 10.1080/17453674.2020.1744852

**Published:** 2020-04-02

**Authors:** Nicola Hewlett-Smith, Rodney Pope, James Furness, Vini Simas, Wayne Hing

**Affiliations:** a Faculty of Health Sciences and Medicine, Bond University, Gold Coast, Australia;; b Allied Health Department, The Wesley Hospital, Brisbane, Australia;; c School of Community Health, Charles Sturt University, Albury, Australia

## Abstract

Background and purpose — Essential for safe and timely hospital discharge, inpatient functional recovery following lower limb arthroplasty is also variable. A previous systematic review reported moderate and conflicting levels of evidence regarding patient-related predictors of inpatient recovery for primary total hip arthroplasty (THA). A systematic review of surgical prognostic factors for inpatient recovery following THA or total knee arthroplasty (TKA) is yet to be undertaken. We identified patient and surgical prognostic factors for inpatient functional recovery following THA and TKA; determined whether inpatient functional recovery varies between these procedures; and established whether validated outcome measures relevant to the patient’s functional requirements for hospital discharge are routinely assessed.

Patients and methods — Critical Appraisal Skills Programme checklists assessed methodological quality, and a best-evidence synthesis approach determined the levels of evidence supporting individual prognostic factors. PubMed, CINAHL, Embase, Scopus, and PEDro databases were searched from inception to May 2019. Included studies examined patient or surgical prognostic factors and a validated measure of post-operative function within 2 weeks of primary, unilateral THA or TKA.

Results — Comorbidity status and preoperative function are supported by a strong level of evidence for TKA. For THA, no strong level of evidence was found for patient-related prognostic factors, and no surgical factors were independently prognostic for either arthroplasty site. Limited evidence supports fast-track protocols in the TKA population.

Interpretation — Preoperative screening and optimization is recommended. Assessment of Enhanced Recovery Pathways using validated outcome measures appropriate for the early postoperative period is warranted.

The International Classification of Function, Disability and Health (WHO 2013) describes the interdependent relationship among function, activity, and participation. Following lower limb arthroplasty, functional recovery is key to the independent performance of fundamental activities of daily living (ADL) such as walking, transferring in and out of bed, and climbing stairs; achieving these milestones is necessary for safe and timely hospital discharge (Shields et al. 1995, Hoogeboom et al. 2015, Poitras et al. 2015). Inability to perform basic ADL increases the patient’s risk of social isolation, falls, and the need for additional resources such as rehabilitation and community services (Poitras et al. 2015).

To promote rapid recovery, multimodal Enhanced Recovery Pathways (ERP) are increasingly used for lower limb arthroplasty (Scott et al. 2013). However, the success of these pathways is primarily assessed via non patient-centric measures including morbidity and mortality, readmission rates, length of stay (LOS), and organizational economic savings (Husted 2012). Functional recovery is linked to discharge destination, longer-term functional outcomes, quality of life (Elbaz et al. 2015), patient satisfaction (Scott et al. 2012), LOS, and associated costs (Husted et al. 2008, 2010, Ibrahim et al. 2013). However, few studies have specifically examined inpatient functional recovery as an outcome following lower limb arthroplasty, using valid measures.

While studies have considered achievement of hospital-specific functional discharge criteria, these constitute neither a standardized nor a validated outcome measure. Whilst LOS may be influenced by wide-ranging factors (Husted et al. 2008, 2010, 2011, Den Hertog et al. 2012, Napier et al. 2013, Elings et al. 2016), inpatient functional recovery is commonly thought to be primarily affected by patient and surgical factors.

Surprisingly, inpatient functional recovery has been investigated by a single systematic review. Based on the results of 2 studies, Elings et al. (2015) reported moderate and conflicting levels of evidence regarding the association between patient-related factors and inpatient functional recovery. Therefore, this systematic review examines the evidence for patient and surgical prognostic factors for inpatient functional recovery following both total hip arthroplasty (THA) and total knee arthroplasty (TKA); determines whether inpatient functional recovery varies between these procedures; and identifies whether validated outcome measures relevant to the patient’s functional requirements for hospital discharge are routinely assessed. The identification of surgical prognostic factors may provide an opportunity to refine ERP, whilst patient-related factors may aid in identifying those at risk of delayed recovery, enabling medical optimization, prehabilitation, and early discharge planning (Oosting et al. 2016).

## Method

The systematic review protocol was registered with PROSPERO (PROSPERO Registration: CRD42019136206), and reporting is in accordance with the PRISMA statement. A comprehensive search of PubMed, CINAHL, Embase, Scopus, and PEDro databases was undertaken on May 31, 2019. The search strategy included key search terms relating to prognostic factors, hip and knee arthroplasty, and function. Subject headings specific to individual databases were utilized, and wildcards employed. No date range or language filters were applied. The PubMed/MEDLINE search strategy is presented in Appendix 1. Reference lists were also examined to capture all potentially eligible publications. Eligibility criteria (Table 1) were established and applied to the search results during initial screening of titles and abstracts. Final selection of articles based on full text review was performed independently by 2 reviewers. Differences were resolved by consensus.

**Table 1.** Eligibility criteria

Criteria	Inclusion	Exclusion
Population	Humans undergoing primary elective total hip or knee arthroplasty	Bilateral total hip or knee arthroplasty Unicompartmental knee arthroplasty Hip joint re-surfacing Arthroplasty performed secondary to fracture (trauma or pathological)
Context	Australian and international studies carried out in public and private hospital settings	Studies not carried out within a public or private hospital Articles not reporting primary research
Language	All languages	Studies where language translation was not possible. However, these studies were noted for completeness, prior to exclusion
Recency of publication	All date periods preceding the search date	
Time period	Studies examining outcomes in the early postoperative period (≤ 2 weeks postoperatively)	Studies where the postoperative time point at which outcome measures were assessed is not specified or was > 2 weeks
Prognostic factors	Studies examining the relationship between 1 or more surgical or patient-related prognostic factors and functional performance or patient-reported outcome measures	Studies where the prognostic factors of interest pertained only to determining the efficacy of a treatment intervention, the specific properties of the prosthesis used or patient genetic, blood, or radiological markers
Outcomes	Studies examining at least 1 validated functional performance or patient reported outcome measure indicating postoperative functional recovery	

Critical Appraisal Skills Programme (CASP) (2019a, 2019b, 2019c) checklists were used to address the methodological quality of the differing study designs and examine external validity, internal validity (bias), internal validity (confounding), and statistical power. To grade methodological quality, a scoring system was devised by the reviewers, and applied to each CASP checklist. Subsequently, Questions 7 and 8 of each checklist, and Question 12 of the Cohort Studies checklist were modified to elicit a “Yes,” “No,” or “Can’t tell” response (Appendix 2). For each checklist question, a “Yes” response scored 1, and a response of “Can’t tell” or “No” scored 0; for questions involving a 2-part answer, parts (a) and (b) were scored separately. Using this system, the CASP checklists for Randomized Controlled Trials, Case Control Studies, and Cohort Studies had a maximum possible score of 11, 12, and 14, respectively. Scores were converted to a percentage and ranges were determined (by the reviewers) to reflect methodological quality as follows: < 30% low quality, 31–65% medium quality, and > 65% high quality. Studies were independently appraised by 2 reviewers, and Cohen’s Kappa (κ) (Cohen 1960) assessed level of agreement; differences were resolved by discussion and consensus.

Extracted data was tabulated, including: study design, context, sample size, demographics, arthroplasty site, prognostic factors, validated measures of postoperative functional recovery, and the time points at which these were assessed. Meta-analysis was not possible due to the methodological heterogeneity of included studies, therefore a best evidence synthesis approach was employed. Evidence levels were ranked as follows: strong evidence is provided by ≥ 2 studies with low risk of bias and by generally consistent findings in all studies (≥ 75% of the studies reported consistent findings); moderate evidence is provided by 1 low risk of bias study and ≥ 2 moderate/high risk of bias studies or by ≥ 2 moderate/high risk of bias studies and by generally consistent findings in all studies (≥ 75%); limited evidence is provided by ≥ 1 moderate/high risk of bias studies or 1 low risk of bias study and by generally consistent findings (≥ 75%); conflicting evidence is provided by conflicting findings (< 75% of the studies reported consistent findings) (Eijgenraam et al. 2018).

**Figure:**
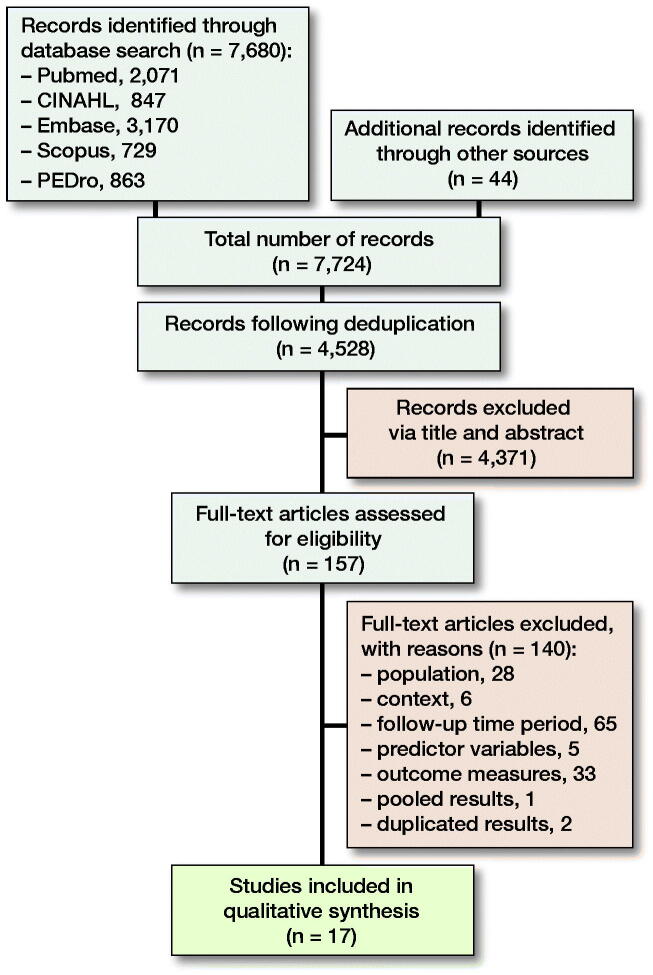


## Results

The search identified 7,724 records and, following screening, 17 studies were included (Figure). These incorporated 1,171 THA and 1,662 TKA procedures. 8 studies investigated THA, 8 TKA, and 1 both procedures (Table 2, see Supplementary data). 12 studies examined patient-related factors (Table 3, see Supplementary data) and 9 studies investigated surgical factors (Table 4, see Supplementary data). Numerous tools evaluated comorbidity status and preoperative function. Postoperative functional recovery was assessed via 14 different validated functional performance and patient-reported outcome measures (PROM). Assessment time points varied significantly between studies within the 2-week postoperative period.

Critical appraisal results are presented in Appendix 3; 7 studies were rated as high methodological quality, 7 as medium quality, and 3 as low quality. There was strong level of agreement between the two reviewers’ judgements (κ = 0.944, p < 0.001). The best-evidence synthesis for prognostic factors for early functional recovery following THA and TKA is presented in Tables 5 and 6 respectively (see Supplementary data).

The heterogeneity of outcome measures employed in the included studies is presented in Appendix 4. Only 7 studies utilized objective outcome measures that assess key functional tasks representative of ADL required for discharge. The Modified Barthel Index (MBI; Shah et al. 1989), Iowa Level of Assistance Scale (ILAS; Shields et al. 1995) and modified Iowa Level of Assistance Scale (mILAS; Oldmeadow et al. 2006) each assess tasks including bed or chair transfers, ambulation, stair climbing, and the amount of assistance required for their achievement. However, the mILAS was further modified (from that published by Oldmeadow et al. 2006) in 2 studies (Elings et al. 2016, van der Sluis et al. 2017) and only partially implemented in all 4 studies where it was assessed, potentially compromising its validity, the generalizability of results and also the ability to compare outcomes between studies. Morri et al. (2016) describe the scoring method for the ILAS inaccurately, casting doubt on the validity of its implementation.

## Discussion

This systematic review examines the evidence for patient-related and surgical prognostic factors for inpatient functional recovery following THA and TKA; determines whether inpatient functional recovery varies depending on arthroplasty site; and identifies whether inpatient functional recovery was assessed using validated outcome measures relevant to the patient’s functional requirements for hospital discharge.

The level of evidence for patient-related prognostic factors and inpatient functional recovery differs between THA and TKA populations. However, associations between timed and observational performance measures of preoperative physical function or comorbidity status (ASA grade) and inpatient recovery was evident for both arthroplasty sites. Conflicting evidence exists for body mass index (BMI) and age as prognostic factors in both arthroplasty populations. The role of sex was supported by limited evidence and conflicting evidence in TKA and THA studies, respectively.

These results contrast to those published by Elings et al. (2015), which (based on 2 included studies) reported moderate-level evidence for preoperative ADL status, female sex, and BMI; and conflicting evidence for increased age, as prognostic factors of delayed inpatient recovery following THA. Moderate-level evidence indicated no association for ASA grade; however, it should be noted this result was based on the findings of a single study. Greater comorbidity (Charnley class C), poorer preoperative functional performance (10-meter walk test, Timed Up and Go [TUG]), and increased age were also confirmed prognostic factors of delayed functional recovery in a further study of 294 THA patients (Oosting et al. 2016), which did not meet inclusion criteria in this review due to some participants undergoing revision surgery.

In summary, preoperative function has consistently been associated with early postoperative function following THA and TKA. The roles of increased comorbidity, older age, sex, and BMI must also be considered. The confirmation of these prognostic factors highlights the need for routine preoperative patient screening. Screening could be implemented conjointly with the decision to proceed to surgery, thus maximizing the preoperative window. Simple performance measures may identify patients potentially at risk of delayed recovery, providing the opportunity for preoperative medical and functional optimization, and prompt discharge planning (Elings et al. 2015, Oosting et al. 2016). Prehabilitation has been demonstrated to improve preoperative function (Swank et al. 2011, Clode et al. 2018) and may successfully be implemented via telerehabilitation (Doiron-Cadrin et al. 2019), thereby capturing patients with reduced access (Westby et al. 2010), whilst avoiding significant cost burden to both patients and healthcare organizations (Fusco and Turchetti 2016).

This review did not identify any surgical factors that were independently prognostic for postoperative functional recovery. Although the overall methodological quality of studies examining surgical factors was of a moderate to high level, sample sizes were small (40–67 participants) in 4 studies, and 3 studies did not report confidence intervals for their results. These results suggest that individual surgical factors may not significantly impact recovery and rather that ERP or Fast-track pathways, which address many aspects of the surgical pathway, are more effective in promoting early functional return. Further research is required to assess the impact of ERP using validated functional outcome measures.

Differences in the pattern of inpatient recovery following THA and TKA require further research. A single study (Kennedy et al. 2006) modelled the recovery pattern for both sites of arthroplasty; however, the methodological quality of this study limits the generalizability of the results. Hierarchical linear modelling was used due to the varied patient numbers and lack of standardization of postoperative time points, and several confounding factors were not accounted for. LOS was reported in 12 studies and appears to range from 2 to 39 days, with 9 studies stating or implying the use of discharge criteria. Due to the heterogeneity of studies with regard to the presence or type of discharge criteria used, how rigorously the discharge criteria were implemented, and when and how functional recovery was assessed, there is insufficient evidence to determine whether inpatient functional recovery differs by arthroplasty site.

Validated tools for assessing short-term postoperative function following lower limb arthroplasty are lacking (Kimmel et al. 2016, Poitras et al. 2016). Currently there is no gold standard for evaluating functional recovery in acute hospital inpatients (Kimmel et al. 2016), which may explain the heterogeneity of outcome measures employed. Several PROMs including the Lower Extremity Function Scale, Knee injury and Osteoarthritis Outcome Score, and WOMAC are appropriate for assessing longer-term functional outcomes as they address more advanced functional activities (Poitras et al. 2016) however these activities are not achieved within the acute recovery phase and are not reflective of ADL required for hospital discharge.

Low to moderate correlations are reported between PROMs and performance measures in the early postoperative period following THA and TKA (Mizner et al. 2011, Poitras et al. 2016). PROMs are subjective and may be influenced by many factors (Poitras et al. 2016), including perceived level of exertion (Mizner at al. 2011), anxiety, and expectations regarding recovery (Salmon et al. 2001b); therefore performance-based measures are necessary to objectively assess actual patient function (Mizner et al. 2011). However, performance measures should be clinically relevant, easily integrated into routine postoperative assessment, appropriate to the time point at which they are assessed, and implemented in a standardized manner to enable evaluation of patient outcomes across organizations. PROMs have been adopted by some National Joint Registries to record longer-term functional outcomes. Similar integration of standardized performance-based assessments could aid in generating a database of early postoperative functional outcomes, thus providing more pertinent information than LOS comparisons.

A strength of this review is the broad search undertaken with few exclusion criteria to ensure all available evidence regarding patient-related and surgical prognostic factors and inpatient functional recovery following THA and TKA was captured. Studies published in all languages were considered for inclusion. There are also several limitations. The heterogeneity of outcome measures assessed and, additionally, the modification, or varied and partial implementation of valid outcome measures (in particular the mILAS) limits the comparison of results between studies. For this reason a meta-analysis was not possible. Not all included studies published results for their early postoperative time points. Moreover, not all studies reported 95% confidence intervals, therefore the significance of some results may be questioned. None of the included studies collected data within the last 4 years, thus the potential impact of more recent surgical advances including muscle-sparing surgical approaches and robotic-assisted surgery has not been assessed. For the purpose of screening, studies where joint ROM was the only postoperative outcome measure examined were excluded. Although a noted contributor, joint ROM alone is not sufficient to enable mobility or the performance of ADL.

## Conclusion

Based on the findings of this review, there is strong level of evidence that comorbidity status determined by ASA grade, and preoperative functional status assessed by the TUG are prognostic factors for inpatient functional recovery following TKA. No strong level of evidence was found for patient-related prognostic factors for inpatient recovery following THA. No surgical factors were found to be independent prognostic factors for inpatient recovery following either THA or TKA; however, limited evidence supports Fast-track protocols in the TKA population. Studies assessing inpatient functional recovery are heterogeneous. Variance in methodological quality, variables examined, outcome measures, and the time points at which they are assessed makes comparison of results difficult. With shorter LOS desirable, preoperative screening is recommended to identify patients at risk of delayed inpatient recovery enabling prehabilitation, medical optimization, and early discharge planning. Valid, standardized performance measures assessing basic functional tasks would assist in objectively determining patient readiness for discharge (Shields et al. 1995), evaluating the success of ERP interventions (Poitras et al. 2016), and enable benchmarking across organizations. Surgical advances in lower limb arthroplasty and their impact on impatient functional recovery are also worthy of investigation.

## Funding and potential conflicts of interest

This research was supported by an Australian Government Research Training Program Scholarship.

Each author certifies that he or she has no commercial associations that might pose a conflict of interest in connection with the submitted article.

## Data statement

N H-S is registered with the data repository Open Science Framework.

## Supplementary Material

Supplemental Material

## Supplementary data

Tables 2–6 and Appendices 1–4 are available as supplementary data in the online version of this article, http://dx.doi.org/ 10.1080/17453674.2020.1744852
