# Supplementary material for: Prognostic factors for inpatient functional recovery following total hip and knee arthroplasty: a systematic review
Source: Acta Orthop. 2020 Apr 2;91(3):313–8. doi: 10.1080/17453674.2020.1744852 (PMC8023898; doi:10.1080/17453674.2020.1744852)
Supplement: Supplemental Material [file IORT_A_1744852_SM5787.pdf]

Supplementary data

Table 2. Study characteristics

Study ID	Study design	Country, number of sites, hospital funding	Arthroplasty type	Population n	Mean age (SD), [a] range	% Female	Prognostic factors	Validated outcome measures
Bade et al. 2014	RCT (2x pooled)	United States, 1, public	TKA	64	64.6 (8.5), NR	50	Patient	TUG
Carli et al. 2010	RCT	Canada, 1, public	TKA	40	71 (NR) 55–85	73	Patient Surgical	Change in 2MWT (POD3–POD1)
Carmichael et al. 2013	RCT	Canada, 1, public	THA	47	60.2_p $(12.8)_p$, NR	39	Surgical	WOMAC
Den Hertog et al. 2012	CCS	Germany, 1, NR	TKA	147	67.4_p $(8.1)_p$, NR	71	Surgical	WOMAC AKSS
Elings et al. 2016	PCS	Netherlands, 2, public	THA	154 [c] 271 [d]	70.5_p $(9.0)_p$, NR	70	Patient	mILAS
Fransen et al. 2018	RCT	Netherlands, 1, public	TKA	50	62.5_p $(8.1)_p$,NR	60	Surgical	TUG SF-12 KOOS
Hoogeboom et al. 2015	PCS	United States, 1, private	TKA	193	65 (10), NR	64	Patient Surgical	mILAS
Ilfeld et al. 2008	RCT	United States, 2, private	TKA	50	65 (NR), 60–70	58	Surgical	6MWT
Kennedy et al. 2006	PCS	Canada, 1 public	THA TKA	152	63.8_p $(9.7)_p$, NR	49	Patient Surgical	TUG 6MWT WOMAC pain WOMAC function
Kennedy et al. 2011	PCS	Canada, 1, public	THA	75	54, 62, 68 (f), NR 52, 60, 70 (m), NR	43	Patient	6MWT LEFS
Kessler and Kafer 2007	PCS	Germany, 1, public	THA	67	63.6 (NR), 37–77	45	Patient Surgical	WOMAC pain WOMAC function WOMAC stiffness
Maiorano et al. 2017	RCS	Italy, 1, private	TKA	353	71.6_p $(8.2)_p$, NR	73	Patient	Change in MBI
Morri et al. 2016	PCS	Italy, 1, public and private	THA	167	60.8 (12.7), NR	62	Patient	ILAS
Ogonda et al. 2005	RCT	United Kingdom, 1, public	THA	219	66.6_p $(10.1)_p$, NR	51	Surgical	mILAS 10mWT SCT (ascending) SCT (descending)
Salmon et al. 2001a	PCS	United Kingdom, 2, public	THA	102	69 (11), NR	62	Patient	10mWT 25mWT
Van der Sluis et al. 2017	PCS	Netherlands, 1, public	TKA	682	70_p $(9.1)_p$, 41–89	73	Patient	mILAS
Wang et al. 1998	PCS	Australia, 1, private	THA	65	71 (NR), 47–87	48	Patient	MBI

[a] Subscript p = pooled; f = female; m = male; NR: not reported.
[b] AKSS: American Knee Society Score, CCS: case control study, ILAS: Iowa Level of Assistance Scale, KOOS: Knee injury and Osteoarthritis Outcome Score, LEFS: Lower Extremity Functional Scale, mILAS: modified Iowa Level of Assistance Scale, MBI: Modified Barthel Index, PCS: prospective cohort study, POD: postoperative day, RCS: retrospective cohort study, RCT: randomized controlled trial, SCT: stair climbing test, SD: standard deviation, SF-12: Short form 12 health survey, THA: total hip arthroplasty, TKA: total knee arthroplasty, TUG: Timed Up and Go, WOMAC: Western Ontario and McMaster Universities Osteoarthritis Index, 2MWT: 2-minute walk test, 6MWT: 6-minute walk test, 10mWT: 10-meter walk test, 25mWT: 25-meter walk test.
[c] Data set A. [d] DATA set B.

Table 3. Patient-related prognostic factors for inpatient functional recovery

Study ID	Arthro-plasty type	Individual prognostic factors	Outcomes assessed	Timepoint of assessment	Association with functional outcome (95% CI)	Methodological quality of study
Bade et al. 2014	TKA	Age<br>Sex<br>TUG [a]<br>6MWT [a]	TUG	POD 2	NR<br>NR<br>ß: –61 (CI –107 to –14) [c]<br>NR	High
Carli et al. 2010	TKA	Age<br>Body weight<br>ASA grade 2<br>CHAMPS [b]<br>WOMAC function ss [b]<br>Knee Society evaluation [b]<br>SF-12 [b]<br>Log 2MWT [a]<br>Total walking time [b] (POD1+ POD 2+ POD3)	Change in 2MWT (POD3–POD1)	POD 1, 2, 3	NR<br>ß: 0.27 (CI 0.07–0.48) [c]<br>ß: –10.6 (CI –20.8 to –0.4) [c]<br>NR<br>NR<br>NR<br>NR<br>ß: 10.3 (CI 3.1–17.5) [c]<br>ß: 0.2 (CI 0.05–0.4) [c]	High
Elings et al. 2016	THA	Age (> 70 years)<br>Sex<br>BMI<br>ASA grade<br>Charnley class<br>WOMAC pain ss [b]<br>WOMAC stiffness ss [b]<br>WOMAC function ss [b]<br>Pain scale [b]<br>Patient-estimated walking capacity (mins) [b]<br>6MWT [a]<br>Chair-rise time [a]<br>Quads power (dynamometer) [a]<br>TUG [a]	mILAS (considered delayed recovery if > 5 days to achieve score of 0–1)	Daily<br><br><br><br><br><br><br><br><br><br><br><br>NR	OR: 1.2 (CI 0.4–3.4) [c]<br>OR: 0.8 (CI 0.2–2.6) [c]<br>OR: 2.2 (CI 0.7–7.4) [c]<br>OR: 1.2 (CI 0.3–4.4) [c]<br>OR: 6.1 (CI 2.2–17.4) [c]<br>NR<br>NR<br>NR<br>NR<br>NR<br>NR<br>NR<br><br>OR: 3.1 (CI 1.1–9.0) [c]<br>AUC = 0.82 (CI 0.7–0.9)	High
Hoogeboom et al. 2015	TKA	Age<br>Sex<br>BMI<br>Marital status<br>Employment status	mILAS (considered delayed recovery if score > 6 on discharge or not attained 0–6 by afternoon POD 2)	Daily per transfer	OR: 1.08 (CI 1.04–1.1) [c]<br>OR: 2.1 (CI 1.1–4.0) [c]<br>OR: 1.1 (CI 1.06–1.2) [c]<br>NR<br>NR<br>(AUC = 0.72: 0.65, 0.80)	High
Kennedy et al. 2006	THA, TKA	Age<br>Sex<br><br>BMI<br>Number of comorbidities<br>TUG [a]<br>6MWT [a]<br>WOMAC pain ss [b]<br>WOMAC function ss [b]	TUG<br>6MWT<br>WOMAC pain<br>WOMAC function	Not standardized 1st time point at hospital DC (generally at 1 week postop)	NR, *ns*<br>$p \le 0.003$ (for TUG and 6MWT at 1 week postop) [c]<br>NR, *ns*<br>NR, *ns*<br>$p \le 0.001$ (TUG at 1 week postop) [c]<br>$p \le 0.001$ (6MWT at 1 week postop) [c]<br>NR<br>NR	Medium
Kennedy et al. 2011	THA	Age<br>Sex<br>BMI<br>LEFS [b]<br>6MWT [a]	6MWT<br>LEFS	Not standardized Appears 1st time point 2 weeks postop	NR, *ns*<br>NR, *ns*<br>NR, *ns*<br>NR, *ns*<br>ß: 0.6 (CI 0.3–0.9) [c] (6MWT, f)<br>ß: 0.6 (CI 0.4–0.7) [c] (6MWT, m)	Low
Kessler and Kafer 2007	THA	Age<br>Sex<br>BMI<br>Affected side<br>WOMAC pain, function and stiffness ss [b]	WOMAC pain, function and stiffness ss	POD 10	OR: –0.01 (CI –0.5–0.5), *ns*<br>OR: –11.9 (CI –22.7 to –1.1) [c]<br>OR: –0.2 (CI –1.2–0.9), *ns*<br>OR: 7.2 (CI –2.7–17.1), *ns*<br>OR: 0.3 (CI 0.1–0.6) [c]	Medium

Table 3 continued

Study ID	Arthro-plasty type	Individual prognostic factors	Outcomes assessed	Timepoint of assessment	Association with functional outcome (95% CI)	Methodological quality of study
Maiorano et al. 2017	TKA	Age<br><br>BMI<br>Charlson index<br>Depressive disorder<br>Other TKA or THA<br>Hemoglobin level<br>MBI [a]	Change in MBI	POD 3 and on discharge from rehab	ß: -0.3 (CI -0.4 to -0.2) [c] (f), *ns* (m)<br>NR, *ns*<br>NR, *ns*<br>NR, *ns*<br>NR, *ns*<br>NR, *ns*<br>ß: –0.8 (CI –0.9 to –0.7) [c] (f)<br>ß: –0.8 (CI –0.9 to –0.8) [c] (m)	Medium
Morri et al. 2016	THA	Age<br>Sex<br>ASA grade	ILAS	During last 24 hours of hospital stay (LOS ≤ 7 days)	ß: –2.9 (CI –4.8 to –1.0) [c]<br>ß: 0.2 (CI 0.1–0.2) [c]<br>NR	Low
Salmon et al. 2001a	THA	POMS [b]<br>WOMAC pain ss [b]<br>WOMAC stiffness ss [b]<br>WOMAC function ss [b]	10mWT<br>25mWT<br>(inpatient recovery scored as number of days to achieve milestones)	Unclear<br>? Daily	NR, *ns*<br>NR, *ns*<br>NR, *ns*<br>NR, *ns*	Low
Van der Sluis et al. 2017	TKA	Age<br>Sex<br>BMI<br>ASA grade<br>Charnley class<br>ISAR score<br>Presence of stairs at home<br>TUG [a]<br>DEMMI [a]	mILAS functional recovery assessed as number of days to achieve score 0–6: considered delayed recovery if took: > 6 days (1st pathway), 4 days (2nd pathway), 3 days (3rd pathway) Data collected over 9 years; pathway updated over this time frame	Daily	OR: 1.06 (CI 1.04–1.09) [c]<br>NR<br>OR: 1.04 (CI 1.00–1.08), *ns*<br>OR: 2.5 (CI 1.5–4.0) [c]<br>NR<br>OR: 1.6 (CI 1.4–2.0) [c]<br>NR<br>OR: 1.10 (CI 1.06–1.15) [c]<br>OR: 0.96 (CI 0.95–0.98) [c]	High
Wang et al. 1998	THA	Age<br>Number of comorbidities<br>MBI [a]<br>Hip strength (dynamometer) [a]	MBI (considered extended LOS if not achieving ≥ 90 by POD 10)<br>Hip strength	POD 3, 5, 8, 10	OR: 3.9 (CI 0.6–27.8), *ns*<br>OR: 2.0 (CI 0.5–7.4), *ns*<br>OR: 6.0 (CI 1.3–28.3) [c]<br>OR: 4.0 (CI 1.0–16.1), *ns*	Medium

[a] Functional performance measure.
[b] Patient-reported outcome measure (PROM).
[c] Indicates significance at 0.05 level.
ASA: American Society of Anesthesiologists, BMI: body mass index, CHAMPS: Community Health Activities Model Program for Seniors, CI: 95% confidence interval, DEMMI: de Morton Mobility Index, f: female sex, ILAS: Iowa Level of Assistance Scale, ISAR: Identification of Seniors At Risk, LEFS: Lower Extremity Functional Scale, MBI: Modified Barthel Index, m: male sex, mILAS: modified Iowa Level of Assistance Scale, *ns*: not significant, NR: not reported, POD: postoperative day, POMS: Profile of Mood States, SF-12: Short form 12 health survey, THA: total hip arthroplasty, TKA: total knee arthroplasty, TUG: Timed Up and Go, WOMAC: Western Ontario and McMaster Universities Osteoarthritis Index (ss = subscale), 2MWT: 2-minute walk test, 6MWT: 6-minute walk test, 10mWT: 10-meter walk test, 25mWT: 25-meter walk test.

Table 4. Surgical prognostic factors for inpatient functional recovery

Study ID	Arthro-plasty type	Individual prognostic factors	Outcomes assessed (within 2-week postoperative time frame)	Timepoint of assessment	Association with functional outcome (95% CI)	Methodological quality of study
Carli et al. 2010	TKA	Anesthesia type:<br>– continuous FNB<br>– periarticular LIA	Change in 2MWT distance (POD3–POD1)	POD 1, 2, 3	NR (except for p = 0.3), *ns*	High
Carmichael et al. 2013	THA	Analgesia type:<br>– pregabalin/celecoxib<br>– placebo/placebo	WOMAC	1-week postop	NR	Medium
Den Hertog et al. 2012	TKA	Fast-track protocol [c]	WOMAC<br>AKSS	POD 5–7	NR (except for p < 0.0001) [a]<br>NR (except for p < 0.0001) [a]<br>Results reported for per protocol cohort only.<br>ITT cohort results NR	High
Fransen et al. 2018	TKA	Fast-track protocol [d]	TUG<br>SF-12<br>KOOS	2 weeks postop	NR (except for p = 0.02) [a]<br>NR, *ns*<br>NR, *ns*	Medium
Hoogeboom et al. 2015	TKA	Blood loss<br>Tourniquet time<br>Surgeon experience<br>Anesthesia type:<br>– continuous FNB<br>– single-shot FNB<br>– general<br>– spinal<br>Analgesia type:<br>– morphine use	mILAS (considered delayed recovery if score > 6 on discharge or not attained 0–6 by afternoon POD 2)	Daily per transfer	OR: 1.00 (CI 0.99–1.01), *ns*<br>OR: 0.99 (CI 0.98–1.01), *ns*<br>OR: 0.36 (CI 0.20–0.66), *ns*<br><br>NR<br>NR<br>NR<br>NR<br><br>OR: 0.95 (CI 0.45–2.01), *ns*	High
Ilfeld et al. 2008	TKA	Anesthesia type:<br>– overnight FNB<br>– 4-day ambulatory FNB	6MWT	Afternoon POD 1	OR: 1.2 (CI 0.7–1.9), *ns*	Medium
Kennedy et al. 2006	TKA, THA	Site of arthroplasty	TUG<br><br>6MWT<br>WOMAC pain<br>WOMAC function	Variable	NR (for TUG and 6MWT at 1 week postop) [b]<br>NR<br>NR [b]<br>NR, *ns*	Medium
Kessler and Kafer 2007	THA	Anchorage of implant<br>Duration of surgery	WOMAC pain, function, and stiffness subscales	POD 10	OR: 4.4 (CI –2.2–11.1), *ns*<br>OR: –0.16 (CI –0.46–0.14), *ns*	Medium
Ogonda et al. 2005	THA	Incision size	mILAS (3x tasks):<br>– supine to sit<br>– sit to stand<br>– walking<br>10mWT<br>SCT (ascending)<br>SCT (descending)	POD 2	 NR (except for p = 0.3), *ns*<br>NR (except for p = 0.3), *ns*<br>NR (except for p = 0.5), *ns*<br>NR (except for p = 1.0), *ns*<br>NR (except for p = 0.8), *ns*<br>NR (except for p = 0.2), *ns*	High

[a] indicates significance at 0.05 level.
[b] indicates significance according to the authors but level of significance not reported or determinable.
[c] Fast-track rehabilitation versus standard rehabilitation: day of surgery mobilization versus mobilization day 2 postop, 2-hour versus 1-hour physiotherapy sessions, group therapy focusing on activities of daily living (ADL) versus individual. Fast-track group also received positive affirmation, encouraged comparison of progress with other patients (competitive care), had known goal length of hospital stay (LOS) and individual case management for discharge planning.
[d] Fast-track protocol vs. regular protocol: spinal vs. general anesthesia, sub-vastus versus medial parapatellar surgical approach, use of patella in-place balancing versus no patella in-place balancing, extent of soft tissue release, no tourniquet versus tourniquet use, no attachments versus standard attachments (patient-controlled analgesia, wound drain, indwelling catheter), use of intraoperative LIA versus no LIA, use of ice packs versus no ice packs, Day 0 versus day 1 mobilization, prn use versus standard use of short-acting opiates.
AKSS: American Knee Society Score, CI: 95% confidence interval, FNB: femoral nerve block, KOOS: Knee injury and Osteoarthritis Outcome Score, LIA: local infiltration anesthesia, mILAS: modified Iowa Level of Assistance scale, ns not significant, NR: not reported, POD: postoperative day, SCT: stair climbing test, SF-12: Short form 12 health survey, THA: total hip arthroplasty, TKA: total knee arthroplasty, TUG: Timed Up and Go, WOMAC: Western Ontario and McMaster Universities Osteoarthritis Index, 2MWT: 2-minute walk test, 6MWT: 6-minute walk test, 10mWT: 10-meter walk test.

Table 5. Best evidence synthesis for total hip arthroplasty prognostic factors

Individual prognostic factors	Study ID, statistical significance	Methodological quality	Overall level of evidence [a]	Association with early functional recovery
Age	Elings et al. [b]<br>Kennedy et al. 2006a, *ns*<br>Kennedy et al. 2011, *ns*<br>Kessler and Kafer, *ns*<br>Morri et al. [b]<br>Wang et al., *ns*	High<br>Medium<br>Low<br>Medium<br>Low<br>Medium	Conflicting	Unclear
Sex	Elings et al. [b]<br>Kennedy et al. 2006 [b]<br>Kennedy et al. 2011, *ns*<br>Kessler and Kafer [b]<br>Morri et al. [b]	High<br>Medium<br>Low<br>Medium<br>Low	Conflicting	Unclear
BMI	Elings et al. [b]<br>Kennedy et al. 2006, *ns*<br>Kennedy et al. 2011, *ns*<br>Kessler and Kafer, *ns*	High<br>Medium<br>Low<br>Medium	Conflicting	Unclear
ASA	Elings et al. [b]	High	Moderate	Yes
Charnley class	Elings et al. [b]	High	Moderate	Yes
Number of comorbidities	Kennedy et al. 2006, *ns*<br>Wang et al., *ns*	Medium<br>Medium	Moderate	No
TUG	Elings et al.[b]<br>Kennedy et al. 2006 [b]	High<br>Medium	Limited	Yes
6MWT	Kennedy et al. 2006 [b]<br>Kennedy et al. 2011 [b]	Medium<br>Low	Limited	Yes
MBI	Wang et al. [b]	Medium	Limited	Yes
WOMAC	Kessler and Kafer [b]<br>Salmon et al. 2001a, *ns*	Medium<br>Low	Conflicting	Unclear
Hip strength	Wang et al., *ns*	Medium	Limited	No
Site of arthroplasty	Kennedy et al. 2006 [c]	Medium	Limited	Yes
Incision size	Ogonda et al., *ns*	High	Limited	No
Anchorage of implant	Kessler and Kafer, *ns*	Medium	Limited	No
Duration of surgery	Kessler and Kafer, *ns*	Medium	Limited	No

[a] The criteria used to rank levels of evidence is described in Methods section.
[b] indicates significance at 0.05 level.
[c] indicates significance according to the authors but level of significance not reported or determinable.
ASA: American Society of Anesthesiologists, BMI: body mass index, MBI: Modified Barthel Index, *ns*: not significant, TUG: Timed Up and Go, WOMAC: Western Ontario and McMaster Universities Osteoarthritis Index, 6MWT: 6-minute walk test.

Table 6. Best evidence synthesis for total knee arthroplasty prognostic factors

Individual prognostic factors	Study ID, statistical significance	Methodological quality	Overall level of evidence [a]	Association with early functional recovery
Age	Hoogeboom et al. [b]<br>Kennedy et al. 2006, *ns*<br>Maiorano et al. [b] for males, females, *ns*<br>van der Sluis et al. [b]	High<br>Medium<br>Medium<br><br>High	Conflicting	Unclear
Sex	Hoogeboom et al. [b]<br>Kennedy et al. 2006 [b]	High<br>Medium	Limited	Yes
BMI	Hoogeboom et al. [b]<br>Kennedy et al. 2006, *ns*<br>Maiorano et al., *ns*<br>van der Sluis et al., *ns*	High<br>Medium<br>Medium<br>High	Conflicting	Unclear
Bodyweight	Carli et al. [b]	High	Limited	Yes
ASA	Carli et al. [b]<br>van der Sluis et al. [b]	High<br>High	Strong	Yes
ISAR	van der Sluis et al. [b]	High	Limited	Yes
Charlson index	Maiorano et al., *ns*	Medium	Limited	No
Number of comorbidities	Kennedy et al. 2006, *ns*	Medium	Limited	No
TUG	Bade et al. [b]<br>van der Sluis et al. [b]<br>Kennedy et al. 2006 [b]	High<br>High<br>Medium	Strong	Yes
2MWT	Carli et al. [b]	High	Limited	Yes
6MWT	Kennedy et al. 2006 [b]	Medium	Limited	Yes
MBI	Maiorano et al. [b]	Medium	Limited	Yes
DEMMI	van der Sluis et al. [b]	High	Limited	Yes
Anesthesia type:<br>– continuous FNB<br>– periarticular LIA	Carli et al., *ns*	High	Limited	No
Anesthesia type:<br>– overnight FNB<br>– 4-day ambulatory FNB	Ilfeld et al., *ns*	Medium	Limited	No
Morphine use	Hoogeboom et al., *ns*	High	Limited	No
Fast-track protocol [d]	Den Hertog et al. [b] (results reported for per protocol cohort only)	High	Limited	Yes
Fast-track protocol [e]	Fransen et al. [b]	Medium		
Site of arthroplasty	Kennedy et al. 2006 [c]	Medium	Limited	Yes
Tourniquet time	Hoogeboom et al., *ns*	High	Limited	No
Blood loss	Hoogeboom et al., *ns*	High	Limited	No
Surgeon experience	Hoogeboom et al., *ns*	High	Limited	No

[a, b, c] See Table 5, [d] See Table 4 [c], and [e] See Table 4 [d].
ASA: American Society of Anesthesiologists, BMI: body mass index, DEMMI: de Morton Mobility Index, FNB: femoral nerve block, ISAR: Identification of Seniors At Risk, LIA: local infiltration anesthesia, MBI :Modified Barthel Index, *ns*: not significant, TUG: Timed Up and Go, 2MWT: 2-minute walk test, 6MWT: 6-minute walk test.

Appendix 1. Search strategy for PubMed/MEDLINE

Database	Search strategy
PubMed/MEDLINE Searching all fields	(predict* OR prognos* OR “Forecasting”[Mesh]) AND ((arthroplast* OR replace* OR prosthes*) AND (lower limb OR hip OR knee) OR (“Arthroplasty, Replacement, Hip”[Mesh] OR “Arthroplasty, Replacement, Knee”[Mesh])) AND (function*)

Appendix 2. CASP checklist modification and scoring for methodological quality

CASP checklist questions were re-worded as follows: Question 7 of CASP checklists (Case Control Study, Cohort Study and Randomized Controlled Trial), “What are the results of this study?” was adjusted to read “Was the treatment effect size worthwhile for the context and population in which it is intended it would be applied?”

Question 8 of CASP checklists (Case Control Study, Cohort Study and Randomized Controlled Trial) “How precise are the results?” was altered to read “Did the confidence limits around the treatment effect indicate that the minimum expected effect would be worthwhile in this context and population?”

Question 12 of CASP checklist (Cohort Study) “What are the implications of this study for practice?” was modified to read “Are there implications of this study for practice?”

Appendix 3. CASP checklist scores for individual studies

Study ID	Checklist	Q1	Q2	Q3	Q4	Q5		Q6		Q7	Q8	Q9	Q10	Q11	Q12	Total score	Percent -age
Bade et al. 2014	Cohort	1	1	1	1	1	1	1	1	1	1	1	0	1	1	13 /14	93
Elings et al. 2016	Cohort	1	1	1	1	1	1	1	1	1	1	1	1	1	1	14 /14	100
Hoogeboom et al. 2015	Cohort	1	1	1	1	0	0	1	1	1	1	1	1	0	1	11 /14	79
Kennedy et al. 2006a	Cohort	1	1	0	1	0	0	1	1	0	0	0	0	0	0	5 /14	38
Kennedy et al. 2011	Cohort	1	0	0	1	0	0	0	1	0	1	0	0	0	0	4 /14	29
Kessler and Kafer 2007	Cohort	1	1	0	1	0	0	1	1	0	1	0	0	0	0	6 /14	43
Maiorano et al. 2017	Cohort	1	0	1	1	0	0	1	0	0	1	0	0	0	0	5 /14	38
Morri et al. 2016	Cohort	1	0	0	0	0	0	0	0	1	1	0	0	1	0	4 /14	29
Salmon et al. 2001a	Cohort	1	0	0	0	0	0	1	1	0	0	0	0	0	0	3 /14	21
Van der Sluis et al. 2017	Cohort	1	1	1	1	1	1	1	1	0	1	1	1	1	1	13 /14	93
Wang et al. 1998	Cohort	1	1	0	1	0	0	0	1	0	1	0	0	1	1	7 /14	50
Carli et al. 2010	RCT	1	1	1	1	1		1		1	1	1	1	1	1	11 /11	100
Carmichael et al. 2018	RCT	1	1	1	1	1		1		0	0	0	1	0	N/A	7 /11	64
Fransen et al. 2018	RCT	1	1	1	0	1		1		0	0	0	0	1	N/A	6 /11	55
Ilfeld et al. 2001	RCT	1	1	1	1	0		1		1	1	0	0	0	N/A	7 /11	64
Ogonda et al. 2005	RCT	1	1	1	1	1		1		1	0	0	1	1	N/A	9 /11	82
Den Hertog et al. 2012	Case control	1	1	1	1	1		1	1	0	0	0	1	1	N/A	9 /12	75

Appendix 4. Outcome measures assessing inpatient functional recovery

Study ID	Outcome measure	Tasks assessed	ICF	Discharge criteria	LOS range, mean (days)
Bade et al. 2014	TUG [a]	Sit to stand<br>Walking speed<br>Ability to turn/change direction	A	Nil	NR
Carli et al. 2010	2MWT [a]	Walking speed<br>Ability to turn/change direction	A	Nil	4–6<br>5
Carmichael et al. 2013	WOMAC [b]	Toilet transfers<br>Bath transfers<br>Donning/doffing socks<br>Sit to stand (from bed and chair)<br>Walking<br>Ability to negotiate stairs<br>Bending to floor<br>Light and heavy domestic duties<br>Car transfers<br>Shopping	A	Nil	NR
Den Hertog et al. 2012	WOMAC [b]<br>AKSS [a]	Toilet transfers<br>Bath transfers<br>Donning/doffing socks<br>Sit to stand (from bed and chair)<br>Walking<br>Ability to negotiate stairs<br>Bending to floor<br>Light and heavy domestic duties<br>Car transfers<br>Shopping<br>Distance walked (AKSS)<br>Stairs (AKSS)<br>Use of walking aid (AKSS)	P, A	Patient confident for discharge. Low-moderate pain. No wound ooze. Independent in ADL (transfers, hygiene). Independently mobile with aid > 250 m.	NR<br>6.75 (FT)<br>13.2 (RP)
Elings et al. 2016	mILAS [a]<br>(scored 0–24)	Supine to sitting<br>Sitting to supine<br>Sit to stand<br>Walking	A	Medically fit mILAS < 1. Necessary care arranged for discharge.	NR
Fransen et al. 2018	TUG [a]<br>SF-12 [b]<br>KOOS [b]	Sit to stand<br>Walking speed<br>Ability to turn/change direction<br>Rolling over<br>Toilet transfers<br>Bath transfers<br>Donning/doffing socks<br>Sit to stand (from bed and chair)<br>Walking on an even surface<br>Ability to negotiate stairs<br>Bending to floor<br>Light and heavy domestic duties<br>Car transfers<br>Shopping	A<br>P, A<br>P, A	Able to walk independently with 2x crutches or a walker. No wound problems. Adequate social support.	NR<br>3.7 (FT)<br>4.7 (RP)
Hoogeboom et al. 2015	mILAS [a]<br>(scored 0–24)	Supine to sitting<br>Sit to stand<br>Walking<br>Stair climbing	A	NR, considered functionally independent if mILAS < 6.	NR<br>2
Ilfeld et al. 2008	6MWT [a]	Walking speed<br>Ability to turn/change direction	A	Adequate analgesia (NRS < 4). No IV opioids ≥ 12 hours. Ability to mobilize > 30 m. Discharge at surgeon's discretion upon fulfilment of criteria and not prior to 10.00 on POD 3.	NR<br>3.5 (placebo)<br>3.6 (Ropiv)

Appendix 4 continued

Study ID	Outcome measure	Tasks assessed	ICF	Discharge criteria	LOS range, mean (days)
Kennedy et al. 2006	TUG [a]<br>6MWT [a]<br>WOMAC [b]	Sit to stand (from bed and chair)<br>Walking speed<br>Ability to turn/change direction<br>Toilet transfers<br>Bath transfers<br>Donning/doffing socks<br>Walking<br>Ability to negotiate stairs<br>Bending to floor<br>Light and heavy domestic duties<br>Car transfers<br>Shopping	A<br>A<br>P, A	NR	NR
Kennedy et al. 2011	6MWT [a]<br>LEFS [b]	Walking speed<br>Ability to turn/change direction<br>Rolling over<br>Bath transfers<br>Donning/doffing socks and shoes<br>Walking (in home)<br>Squatting<br>Car transfers<br>Light and heavy domestic duties<br>Lifting objects (from floor)<br>Sitting and standing for 1 hour<br>Walking (2 blocks)<br>Walking 1 mile<br>Work<br>Hobbies/sports<br>Running on even and uneven ground<br>Making sharp turns whilst running<br>Hopping	A<br>A	NR	5–7<br>NR
Kessler and Kafer 2007	WOMAC [b]	Toilet transfers<br>Bath transfers<br>Donning/doffing socks<br>Sit to stand (from bed and chair)<br>Walking<br>Ability to negotiate stairs<br>Bending to floor<br>Light and heavy domestic duties<br>Car transfers<br>Shopping	P, A	NR	10–14<br>10.2
Maiorano et al. 2017	MBI [a]	Bed to chair transfers<br>Ability to mobilize (± walking aid)<br>Ability to negotiate stairs<br>Dressing<br>Grooming<br>Feeding<br>Bathing<br>Toileting<br>Bladder and bowel continence	P, A	Independent bed to chair transfers. Negotiating stairs with crutches. Managing personal care	NR<br>15.3 (f)<br>13.4 (m)
Morri et al. 2016	ILAS [a] (scored 0–50) NB: should generate score of 0–36 for 6 tasks	Supine to sitting<br>Sit to stand<br>Walking<br>Stair climbing<br>Gait speed<br>Type of walking aid used	A, E	NR	NR<br>5.6<br>(19 patients excluded as LOS > 7 days)

Appendix 4 continued

Study ID	Outcome measure	Tasks assessed	ICF	Discharge criteria	LOS range, mean (days)
Ogonda et al. 2005	mILAS [a] (scored 0–18)<br>10mWT [a]<br>SCT (ascending and descending) [a]	Supine to sitting<br>Sit to stand<br>Walking<br>Walking speed<br>Speed and ability to negotiate stairs	A	NR	2–13<br>3.65 (SI)<br>2–22<br>3.68 (RI)
Salmon et al. 2001a	10mWT [a]<br>25mWT [a]	Walking speed	A	Not stated, implied: independent with walking aid, negotiating stairs (if required)	NR<br>19
Van der Sluis et al. 2017	mILAS [a] (scored 0–30)	Supine to sitting<br>Sitting to supine<br>Sit to stand<br>Walking<br>Stair climbing	A	Not stated, implied: considered functionally recovered once mILAS ≤ 6	NR
Wang et al. 1998	MBI [a]	Bed to chair transfers<br>Ability to mobilize (± walking aid)<br>Ability to negotiate stairs<br>Dressing<br>Grooming<br>Feeding<br>Bathing<br>Toileting<br>Bladder and bowel continence	A	MBI ≥ 90, unclear if any other criteria used	5–39<br>NR

[a] Functional performance outcome measure.
[b] Patient-reported outcome measure.
ADL: activities of daily living, AKSS: American Knee Society Score, f: female, FT: fast-track protocol, ICF: International Classification of Function, Disability and Health, ILAS: Iowa Level of Assistance Scale, KOOS: Knee injury and Osteoarthritis Outcome Score, LEFS: Lower Extremity Functional Scale, m: male, MBI: Modified Barthel Index, mILAS: Modified Iowa Level of Assistance Scale, NR: not reported, NRS: numerical rating scale, RI: routine incision, POD: postoperative day, Ropiv: ropivacaine group, RP: regular protocol, SCT: stair climbing test, SF-12: Short form 12 health survey, SI: small incision, TUG: Timed Up and Go, WOMAC: Western Ontario and McMaster Universities Osteoarthritis Index, 2MWT: 2-minute walk test, 6MWT: 6-minute walk test, 10mWT: 10-meter walk test, 25mWT: 25-meter walk test.
